# 834. Characterization of Heavily Treatment Experienced HIV-1 Infected Clinical Trial Participants Infected with SARS-CoV-2 COVID 19: Fostemsavir BRIGHTE Phase 3 Clinical Trial

**DOI:** 10.1093/ofid/ofab466.1030

**Published:** 2021-12-04

**Authors:** Shiven Chabria, Stephane De Wit, Amy Pierce, Bronagh M Shepherd, Michael Warwick-Sanders, Fangfang Du, Marcia Wang, Andrew Clark, Peter Ackerman

**Affiliations:** 1 ViiV Healthcare, Branford, CT; 2 CHU Saint-Pierre, Université Libre de Bruxelles, Brussels, Brussels Hoofdstedelijk Gewest, Belgium; 3 GlaxoSmithKline, Brentford, UK; 4 GSK, London, UK; 5 Temple University, Chesterbrook, PA

## Abstract

**Background:**

BRIGHTE is an ongoing global study evaluating the gp120 attachment inhibitor fostemsavir (FTR) in heavily treatment-experienced (HTE) adults with multidrug resistant (MDR) HIV-1 unable to form a viable antiretroviral (ARV) regimen. An estimated 2 million people living with HIV-1 have been infected with SARS-CoV-2. Those with HIV viremia and/or low CD4+ counts are at increased risk of serious adverse outcome. We describe the reported COVID cases in a clinical trial population of people living with MDR HIV and immune suppression.

**Methods:**

At the start of the COVID pandemic, all ongoing BRIGHTE subjects had achieved ≥ 192 weeks on FTR and optimized background ARVs; results through Week 96 were presented previously. Investigators used WHO guidelines for COVID diagnosis and reported exposure, testing results and symptom presence.

Figure 1. BRIGHTE Study Design

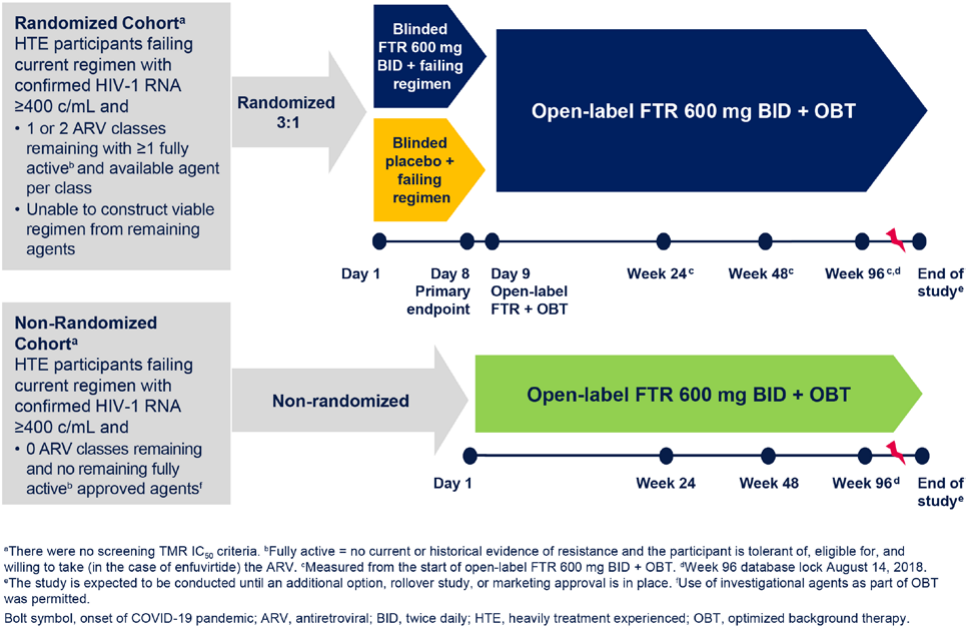

**Results:**

371 subjects [272 Randomized Cohort (RC), 99 Non-Randomized Cohort (NC)] were enrolled; 44% were ≥ 50 years of age and 86% had an AIDS history. Median CD4+ count at study start of was 80 cells/mm^3^ (IQR 11–202); 30% with ≤ 20 cells/mm^3^. 250 subjects remained in BRIGHTE at pandemic start. By April 2021, 17 subjects (14 RC, 3 NC) had confirmed COVID infection (positive PCR test). Severity was Grade 1-3, all cases resolved with no deaths. Six subjects were hospitalized (Table 1); most recent CD4+ count prior to COVID were 293-1641 cells/mm^3^ and 5/6 subjects were virologically suppressed. Treatments often included prophylactic anticoagulants and supplemental oxygen; no cART changes were made. The remaining 11/17 confirmed cases were managed outpatient. Five more subjects had suspect COVID not confirmed by PCR and 2 subjects had negative PCR tests.

Table 1. Characterization of Participants with Serious AEs of Confirmed COVID-19 Infections – All Hospitalizations

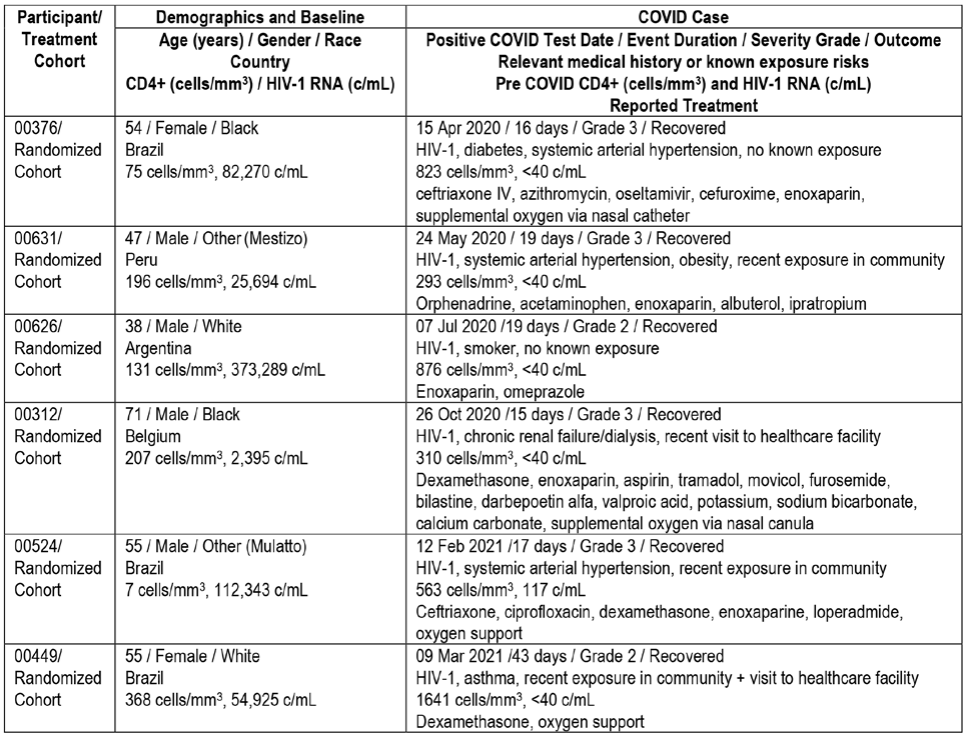

**Conclusion:**

A total of 22/250 COVID-19 cases (17 confirmed, 5 unconfirmed) have been reported in BRIGHTE. Outcomes were reassuring with no deaths or known persistent sequelae, despite having advanced HIV and comorbid diseases at baseline associated with poorer COVID outcomes. Outcomes may have benefitted from immunologic improvement during the trial.

**Disclosures:**

**Shiven Chabria, MD**, **Viiv Healthcare** (Employee) **Stephane De Wit, MD**, **Gilead** (Grant/Research Support)**Janssen** (Grant/Research Support)**Merck Sharpe & Dohme** (Grant/Research Support)**ViiV Healthcare** (Grant/Research Support) **Amy Pierce, BS**, **GlaxoSmithKline** (Shareholder)**ViiV Healthcare** (Employee) **Bronagh M. Shepherd, PhD**, **GlaxoSmithKline** (Employee, Shareholder) **Michael Warwick-Sanders, BM BSc DPM MFPM**, **GSK** (Employee) **Marcia Wang, PhD**, **GlaxoSmithKline** (Employee, Shareholder) **Andrew Clark, MD**, **GlaxoSmithKline** (Shareholder)**ViiV Healthcare** (Employee) **Peter Ackerman, MD**, **GSK/ViiV Healthcare** (Employee, Shareholder)

